# Survey data on home gardeners and urban gardening practice in Pune, India

**DOI:** 10.1016/j.dib.2019.104652

**Published:** 2019-10-11

**Authors:** Ingo Zasada, Siddhartha Lawrence Benninger, Meike Weltin

**Affiliations:** a Leibniz Centre for Agricultural Landscape Research, Germany, Eberswalder Str. 84, 15374 Müncheberg, Germany; b Centre for Development Study and Activities (CDSA), Pune, India, Survey No. 58 & 49/4, Bavdhan Khurd, 411021, Pune, India

**Keywords:** Urban agriculture, Gardening practice, Pune, India, Sustainability

## Abstract

The role of home gardens as productive green spaces in emerging megacities has so far been neglected when addressing pathways for sustainable urban development. This data article provides quantitative data from a questionnaire survey on gardening practice among home gardeners in Pune, one of the fastest growing cities in India and the Asian-Pacific region. Data include growing decisions and food production, fertilization, treatment of pests or irrigation as well as the cultural and recreational use of the garden. The survey also covered sociodemographic background information on the respondents and their gardening motivations. The data were used for a research article to build indicators for economic, environmental and socio-cultural sustainability dimensions of urban agriculture and analyze gardeners' characteristics that lead to increased sustainability outcomes entitled “Home Gardening Practice in Pune (India), the Role of Communities, Urban Environment and the Contribution to Urban Sustainability”[1]. The data and questionnaire are provided at the Open Research Data repository at the Leibniz-Centre for Agricultural Landscape Research (ZALF), Germany (https://doi.org/10.4228/ZALF.DK.109). The data offer an empirical baseline to conduct studies on the interrelationships of gardeners' motivations, gardening practice and the value and outcomes of home gardening in an accelerated urbanization process.

**Table:** Specifications Table

Subject area	*Agricultural sciences*
More specific subject area	*Food Sciences*
Type of data	*Table*
How data was acquired	*Questionnaire survey*
Data format	*Raw data and questionnaire*
Experimental factors	*The survey containing closed questions covers 111 home gardeners (43 with a rooftop-terrace garden, 65 with a backyard-kitchen garden, 3 other gardens).*
Experimental features	*The data was gathered by direct on-site interviews in Pune, India, between January and May 2014. Respondents were recruited via snowball sampling starting with members of the local gardening club INORA (*www.inora.in*).*
Data source location	*Pune, India*
Data accessibility	*Open Research Data. The platform for freely accessible landscape research data at the Leibniz Centre for Agricultural Landscape Research,*https://doi.org/10.4228/ZALF.DK.109.
Related research article	*I. Zasada, M. Weltin, F. Zoll, S.L. Benninger, Home Gardening Practice in Pune (India), the Role of Communities, Urban Environment and the Contribution to Urban Sustainability. Urban Ecosystems.*

**Table:** 

**Value of the Data** • This unique dataset about gardening practice in Pune representing a growing megacity is of particular value as empirical evidence in urban gardening is often lacking or only of qualitative nature [2,3]. • The data give insights into a specific and widespread urban gardening scene, which is characterized by organic management principles and the closing of resource cycles. • Detailed information is provided on gardening practice and the socio-economic background of home gardeners including their motivations. Thus descriptive and regression-based analyses have the potential to reveal determinants of gardeners' behavior. • The data may shed light on the multi-faceted relationships between socioeconomic variables, attitudes and the type of practices used by home gardeners which promote or hamper home-gardening sustainability. • In terms of policymaking and planning, key factors can be extracted to design home-gardening community-based strategies and interventions relevant to local food consumption, availability and consumption of certain nutrients, recreation and building social capital, among others. • The data can be used for but are not limited to studies on sustainability assessments, gardeners' decision-making, cultivation practices, food production, and socio-cultural habits and traditions.

## 1 Data

The data consist of a data file of 111 observations corresponding to the interviewed home gardeners and 235 variables. Each variable is connected to a question in the survey questionnaire. This questionnaire is provided with the data at the Open Research Data repository at the Leibniz-Centre for Agricultural Landscape Research (ZALF), Germany (https://doi.org/10.4228/ZALF.DK.109). The questionnaire is divided in two parts: 1) “Biographic/Demographic Information” and 2) “Growing Food/Gardening”. The first part of includes biographic and socio-economic information of the gardener and the household, community interaction, motivation and restrictions. The second part concentrates on the gardening practice, including cropping types, cropping pattern, gardening vmanagement, inputs, gardening facilities and specific activities. Further information is given on the urban environment and building situation of the garden. The data has already been used for a sustainability assessment of home gardens [1].

All variables were summarized according to a common coding scheme. The data consist of variables with binary response options (mostly coded as 1 = “yes” and 0 = “no”), multivariate response options coded according to the number of alternatives and metric response options. Missing values were coded as 99. All codes and variable names are provided in the survey questionnaire with the respective question.

## 2 Experimental design, materials, and methods

### 2.1 Questionnaire development

Guided by the subject of investigation and research questions about the practice of home gardening in urban housing neighborhoods and the gardening community, the questionnaire has been developed after a series of in-depth interviews with members of a local gardening club in Pune INORA (http://inora.in) about specifics and peculiarities of the local gardening practices. The questionnaire has also been pre-tested with a small group of gardeners from the INORA gardening club. We based all steps on established guidelines for questionnaire development according to Refs. [4,5].

### 2.2 Sampling procedure and data collection

The survey was targeted to Pune which is representative for a fast growing megacity in India and the Asian Pacific region as the population almost doubled between 2011 and 2018 [6]. Many green areas got lost in this process [7]. Thus urban gardening is increasingly important to provide functional green areas valuable for the sustainable development of the city. The survey focused on residential areas of Pune. Due to their specific characteristics, which either do not allow for home gardening or yield other types of urban food production, we have excluded informal housing areas as well as state institution areas. The areas where the survey was conducted covered districts of all different kinds of urban structure occurring in major cities in India, including the old town, pre-independence areas, post-independence areas, and post-economic liberalization areas. We focused on terrace-rooftop gardens and backyard-kitchen gardens because these represent the predominant types of home gardening activities in Indian metropolitan areas as identified in the in-depth interviews.

As official data on home gardening in Pune does not exist, the sampling followed a snowball method starting with members of the INORA gardening club as a key organization in the field of home gardening in Pune as well as a social media group (Facebook group named “Pune Gardeners”). After conducting the interviews, the interviewees have been asked for other home gardeners. In general, there was a high willingness to participate among the gardeners, who have been approached. A predominant share of potential interviewees finally participated in the interviews, although due to the specifics of the process – especially the fact that interviewees were collected through two different channels – it was not possible to accurately track response rates.

The chosen survey mode has been face-to-face interviews following an established methodology [4,5]. The interviews took place between January and May 2014. Gardeners have been contacted via phone, clarifying suitability and willingness to be interviewed. The interviews have been carried out on-site with the help of six commonly instructed interviewers, either in English or Marathi language following the structured questionnaire. All interviewers were bilingual. Translations of the questionnaire and data entries have been cross-checked by a native speaker of both languages also doing research in the field of urban gardening. The effect of the dual language use is thus negligible. Data have been anonymized.
